# Awareness of toxoplasmosis among postpartum women: a cross-sectional study in Morocco

**DOI:** 10.11604/pamj.2022.41.282.31049

**Published:** 2022-04-07

**Authors:** Kenza Hattoufi, Kamal El Bissati, El Bachir Adlaoui, Hassan Aguenaou, Aicha Kharbach, Amina Barkat

**Affiliations:** 1 National Reference Center in Neonatology and Nutrition, Children’s Hospital, University Hospital Centre IBN SINA, Rabat, Morocco,; 2 Mohammed V University in Rabat, Research Team on Health and Nutrition of Mother and Child, Faculty of Medicine and Pharmacy, Rabat, Morocco,; 3 The University of Chicago School of Medicine, Chicago, IL, USA,; 4 University Mohamed VI Polytechnic (UM6P), Coalition Center of Innovation and Prevention of Epidemics in Morocco (CIPEM), Benguerir, Morocco,; 5 National Institute of Hygiene, Rabat, Morocco,; 6 Ibn Tofail University-National Center for Energy Sciences and Nuclear Techniques (CNESTEN), Joint Research Unit in Nutrition, Health and Environnement, RDC-Nutrition African Regional Cooperative Agreement for Research, Development and Training Related to Nuclear Science and Technology/International Atomic Energy Agency (AFRA/IAEA), Rabat-Kenitra, Morocco,; 7 Gynecology-Obstetrics and Endocrinology Department, Maternity Souissi, University Hospital Center IBN SINA, Rabat, Morocco

**Keywords:** Awareness, knowledge, parturient, prevention, toxoplasmosis

## Abstract

**Introduction:**

awareness and knowledge of toxoplasmosis are particularly important, as an intervention point for the management of the disease. The aim of this study was to evaluate the awareness and knowledge regarding toxoplasmosis in a sample of postpartum Moroccan women.

**Methods:**

this was a cross-sectional descriptive survey carried out among 320 parturient at the National Reference Center for Neonatology and Nutrition at the Children's Hospital of Rabat.

**Results:**

of 320 parturient women responding to the survey, 227 (71%) had never heard about toxoplasmosis. While 18.1% of parturient stated knowing the transmission routes for toxoplasmosis. Regarding the transmission route, 53 (16.6%) women pointed at a domestic cat and 31 (9.7%) at eating raw or undercooked meat. Out of all participants, 60 (18.8%) women said they had received prevention advice during their pregnancy. The great majority (90%) of participants were unaware of the severity of the congenital infection. None of the participants showed a high level of knowledge about transmission routes, measures of prevention, and severity of CT.

**Conclusion:**

according to our survey, we observed that the majority of participants had never heard any information about toxoplasmosis. It is, therefore, necessary to educate women of childbearing age and pregnant women about the disease, especially concerning the transmission route and the prevention of infection and primary infection in non-immune women.

## Introduction

Toxoplasmosis is a cosmopolitan parasitic zoonosis, very common in Europe, South America, and Africa. The frequency and severity of toxoplasmosis remain a significant health threat. The disease is caused by the apicomplexan parasite *Toxoplasma gondii (T. gondii)*, which infects approximately one third (25-30%) of the human population worldwide [1,2]. In humans, infection with *T. gondii* is often unrecognized or causes a range of clinical symptoms from self-limited, mild to severe illness [3]. The multiplication of the parasite is limited by the immune response, but it can persist in many tissues (muscles and brain) throughout the life of the human host [4,5]. The infected cat, which is a transmission vector of toxoplasmosis disease, sheds millions of oocysts in their feces, then, the process of sporogony occurs in the external environment after a few days following the excretion [6]. The oocysts can resist for long periods of time in humid and dry conditions [7].

The human may be acquired the infection through the ingestion of tissue cysts in undercooked contaminated meat or by the ingestion of oocysts from contaminated food, hands, soil, as well as, untreated water contaminated with cat feces. As well, *T. gondii* may be transmitted vertically from the mother to the fetus by the passage of the parasite via the placenta [8,9]. Primary acquired maternal infection by *T. gondii* during pregnancy can lead to congenital toxoplasmosis (CT), which may cause fetal and/or neonatal complications [9,10]. The risk related to mother-to-child transmission of *T. gondii* is significantly variable depending on the term of pregnancy. The severity of clinical signs of CT is related to the occurrence of infection during the first trimester of pregnancy; it can lead to spontaneous abortion or severe fetopathy. While the risk of vertical transmission of the parasite increases according to the advancing gestational age. Therefore, the highest rates of transmission occur in the last months of pregnancy, but the infection is usually asymptomatic at this stage. The risk of transmission varies from 6% in the first trimester to around 80% during the third trimester [9,11,12]. The classic triad of CT is chorioretinitis, intracranial calcifications, and hydrocephalus [13].

The global incidence of CT is estimated at 190,100 cases; therefore, early identification of seroconverted women and antenatal treatment reduces their severity [13,14]. In Morocco, the establishment of a rational approach to managing CT was the subject of recent studies that were performed in collaboration with the National Institute of Hygiene, Rabat. This concludes with the implementation of the first International Collaborative POC-Prenatal Screening Consortium in Morocco to evaluate a Toxoplasmosis Point of Care (POC)-Prenatal screening using a point of care testing using whole blood directly from a fingerstick [15]. These are in favor of the implementation of a national screening program for pregnant women. To manage CT, programs must be introduced to screen, trait, and educate women. It is, moreover, of interest to know the women´s level of knowledge about toxoplasmosis. This study was carried out among postpartum Moroccan women during postpartum, aimed to evaluate the knowledge and awareness regarding toxoplasmosis.

## Methods

**Study design:** we used a cross-sectional survey to explore awareness about toxoplasmosis among post-parturient women. Data collection took place between February 2018 and May 2018.

**Study setting:** this study was conducted in the city of Rabat at the National Reference Center for Neonatology and Nutrition situated in the Children's Hospital of Rabat.

**Participants:** the target population was postpartum women aged above 18 years, who are presented to the Neonatology Department for a medical examination of their newborns were asked to participate in the study. All participants were recruited after giving verbal consent. We excluded women who were non-Arabic speakers.

**Sample size determination:** sample size of 320 women was calculated using the formula


$$n=\frac{Z^{2}xp(100-p)}{X^{2}}$$

Z represents the standard normal deviates set at 1.96, which corresponds to a 95% confidence level. X represents a 5% margin of error. p is a 25% estimated prevalence of awareness about toxoplasmosis. We assuming a non-response rate of 10%. In order to make up for attrition an anticipated 90% (0.9) response rate was used. Then final sample size = 288/(1-0.1) = 288/0.9= 320. Participants were selected by convenience sampling. Only postpartum women were eligible.

**Data collection:** the data were collected face-to-face using a questionnaire was composed of four sections: socio-demographic characteristics: age, residence, educational level, profession, marital status, gravidity and parity, monitoring of pregnancy and establishment of pregnancy monitoring; food, hygiene, and lifestyle data; awareness about toxoplasmosis: transmission routes, measures of prevention, and severity of congenital infection; serological status. The question was answered orally and recorded by the researcher. The women's serological status has been confirmed by using the health booklet.

**Evaluation of the level of knowledge:** the evaluation of the level of knowledge regarding the transmission routes, the measures of prevention, and the severity of congenital infection was carried out according to the following levels: high level of knowledge: whether the participants are knowledgeable regarding all transmission routes, measures of prevention and consequences of CT; medium level of knowledge: whether the participants are knowledgeable on at least three transmission routes, three measures of prevention, and three consequences of CT; poor level of knowledge: whether the participants are knowledgeable about at least three transmission routes, measures of prevention, and consequences of CT.

**Statistical analysis:** it was performed using the statistical software the Package for the Social Sciences (SPSS.19). No missing data was found in the questionnaire. A descriptive study of quantitative Gaussian distribution variable (age of the participants) was performed using mean and standard deviation. Qualitative variables were expressed using frequencies and percentages. Awareness of toxoplasmosis according to the socio-demographic characteristics of surveyed women were compared using the χ^2^ test and Fisher test according to the conditions of application of each test. A p-value < 0.05 was considered statistically significant.

## Results

**Socio-demographic characteristics of surveyed women:** total of 320 women was approached to participate in this study. The acceptance rate was 100% as no one refused to participate in the study. The general characteristics of the included women are provided in Table 1. The average age of the participants was 30 ± 7 years (range 18 to 46 years). The majority of women (56.2%) were in the age range between 18 and 30 years old. Multiparous women represented 54.4%. Only 10.3% of the participants were from rural areas. Regarding the education level of the participants, 26.6% were illiterate, and only 15.3% have a university-level degree. The majority of women were married, while three (0.9%) were single mothers. Housewives accounted for 91.6%.

**Table 1:** socio-demographic and lifestyle characteristics of the study participants (N=320)

Characteristics (N=320)	Frequency (%)
**Age (Years)**	
18-30	180 (56.2)
31-40	125 (39.1)
>40	15 (4.7)
**Residence**	
Urban	249 (77.8)
Suburban	38 (11.9)
Rural	33(10.3)
**Educational level**	
Illiterate	85 (26.6)
Primary	66 (20.6)
Secondary	120 (37.5)
Higher education	49 (15.3)
**Profession**	
Housewife	293 (91.6)
Salaried	27(8.4)
**Marital status**	
Married	316 (98.8)
Single	3 (0.9)
Divorced	1 (0.3)
**Gravidity**	
Primigravida	118 (36.9)
Multigravida	202 (63.1)
**Parity**	
Primiparous	146 (45.6)
Multiparous	174 (54.4)
**Monitoring of pregnancy**	
Well monitoring	292 (91.3)
Poor monitoring	19 (5.9)
No monitoring	9 (2.8)
**Establishment of pregnancy monitoring**	
Private	135(43.4)
Public	111 (35.7)
Both	65 (20.9)
**Lifestyle and hygiene**	
Cat at home	39 (12.2)
Disinfect the litter box	18 (46.2)
Contact with stray cats	68 (21.3)
Gardening and/or agricultural activities possession of refrigerator	35 (10.9) 297 (92.8)
Handwashing before meals	309 (96.6)
Consumption of raw vegetables	310 (96.9)
Vegetables and/or fruit washing in bleach	26 (8.1)
Consumption of raw or undercooked meat	12 (3.8)
Consumption of untreated water	37 (11.6)
Consumption of raw milk	116 (36.3)
Eating foods prepared outside (fast food)	207 (64.7)

**Lifestyle and hygiene:** we report that 12.2% of the participants have a cat at home, and 21.3% have indicated the presence of stray cats around their house. The women doing gardening activities accounted for 10.9%, none of them used gardening gloves. Most of the participants (96.9%) eating raw vegetables, while only 3.8% said they ate raw or undercooked meat. Raw milk is consumed by 36.3% of the women, whereas, untreated water is consumed by 11.6%. An additional, 64.7% of participating women eating meals prepared outside the home (Table 1).

**Awareness about toxoplasmosis:** regarding women's awareness regarding toxoplasmosis, 93 (29%) reported knowing about the disease; they had heard or read information about it, while; 71% had never heard about toxoplasmosis. Only 18.1% of participants stated knowing the transmission routes for toxoplasmosis. The most frequent answer regarding the transmission of *T. gondii* given by the participants was contact with cats (16.6%). Sixty women (18.8%), said they had received prevention advice during their pregnancy. Among them, 68% received the information from the medical doctor; 13.3% searched for the information on the internet, while two women received this information during their studies and one woman said she received it from a nurse. The great majority (90%) of participants were unaware of the severity of the fetal damage (Table 2). None of the participants showed a high level of knowledge about transmission routes, measures of prevention, and severity of CT. Participants who have a medium level of awareness about the transmission routes, measures of prevention, and severity of CT accounted for 36.2%, 36.7%, 3.1% respectively (Table 3). The difference in the awareness about toxoplasmosis by age group, residence location, marital status, gravidity, and parity showed no statistical significance. Women who have a university-level degree had better awareness than the others (p <0.001). Salaried women had better awareness than housewives (p=0.001). Women who were well monitored during pregnancy had better awareness than those who were poor or not monitored (p= 0.008) (Table 4). The serological status of 129 women had been verified by consulting their health booklet; among them, 41% were immunized.

**Table 2:** awareness and knowledge about toxoplasmosis

Characteristics (N=320)	Frequency (%)
**Awareness about toxoplasmosis**	
Aware	93 (29.0)
Unaware	227 (71.0)
Means of transmission of toxoplasmosis*	
I don't know	262 (81.9)
Contact with cat	53 (16.6)
Contact with the soil	8 (2.5)
Drinking untreated water	1 (0.3)
Eating raw or undercooked meat	31 (9.7)
Eating unwashed vegetables and fruits	26 (8.1)
Eating outside	2 (0.6)
Drinking raw milk	2 (0.6)
Measures of toxoplasmosis prevention*	
I don't know	260 (81.0)
Avoid contact with cats	44 (13.8)
Avoid contact with soil	4 (1.3)
Consumption of well-cooked meat	26 (8.1)
Wash vegetables and fruits well	28 (8.8)
Avoid eating out	6 (1.9)
Wash hands well	3 (0.9)
keep a good level of hygiene	2 (0.6)
Avoid consumption of raw milk	1 (0.3)
Complications of congenital toxoplasmosis*	
I don't know	288 (90.0)
Malformation	25 (7.8)
Miscarriage	4 (1.3)
Hydrocephalus	2 (0.6)
Prematurity	1 (0.3)
Ocular involvement	4 (1.3)
Deafness	1 (0.3)
Brain damage	2 (0.6)

CT: congenital toxoplasmosis except for “I don't know” answer, the other categories of data are not mutually exclusive

**Table 3:** level of awareness regarding transmission routes, measures of prevention and severity of CT

Characteristics (N=320)	Frequency (%)
**Transmission routes**	
Medium level of knowledge	21 (36.2)
Poor level of knowledge	37 (63.8)
**Measures of prevention**	
Medium level of knowledge	22 (36.7)
Poor level of knowledge	38 (63.3)
**Severity of CT**	
Medium level of knowledge	1 (3.1)
Poor level of knowledge	31 (96.9)

CT: congenital toxoplasmosis

**Table 4:** factors associated with toxoplasmosis awareness on univariate analysis (N=320)

Characteristics (N=320)	Aware frequency (%) N = 93	Unaware frequency (%) N= 227	p- value
**Age (Years)**			
18-30	51 (28.3)	129 (71.7)	0.631
31-40	36 (28.8)	89 (71.2)	
>40	6 (40.0)	9 (60.0)	
**Residence**			
Urban	77 (30.9)	172 (69.1)	0.278
Suburban	7 (18.4)	31 (81.6)	
Rural	9 (27.3)	24 (72.7)	
**Educational level**			
Illiterate	11 (12.9)	74 (87.1)	<0.001
Primary	18 (27.3)	48 (72.7)	
Secondary	34 (28.3)	86 (71.7)	
Higher education	30 (61.2)	19 (38.8)	
**Profession**			
Housewife	77 (26.3)	216 (73.7)	0.001
Salaried	16 (59.3)	11 (40.7)	
**Marital status**			
Married	93 (29.4)	223 (70.6)	0.436
Single	0.0	3 (100.0)	
Divorced	0.0	1 (100.0)	
**Gravidity**			
Primigravida	37 (31.4)	81 (68.6)	0.490
Multigravida	56 (27.7)	146 (72.3)	
**Parity**			
Primiparous	42 (28.8)	104 (71.2)	0.915
Multiparous	51 (29.3)	123 (70.7)	
**Monitoring of pregnancy**			
Well monitoring	92 (31.5)	200 (68.5)	
Poor monitoring	1 (5.3)	18 (94.7)	0.008
No monitoring	0.0	9 (100.0)	
**Establishment of pregnancy monitoring**			
Private			
Public	57 (42.2)	78 (57.8)	<0.001
Both	20 (18.0)	91 (82.0)	
	16 (24.6)	49 (75.4)	

## Discussion

An evaluation of awareness concerning toxoplasmosis was carried out among 320 Moroccan women. This study showed that about 71% of participants had never read, heard or seen information regarding toxoplasmosis. This finding places unimmunized women in a high-risk group, as the lack of information could allow exposure to the parasite throughout subsequent pregnancies. Several studies were consistent with our results, they reported a lack of knowledge and awareness regarding toxoplasmosis [16-19]. While Jones *et al*. reported that 48% of surveyed women indicated that they heard or seen information about the disease [20]. As per, Ogunmodede *et al*. higher levels of education have a significant association with knowledge of the disease. This is concordant with our findings, which revealed that 61.1% of the women who have a higher level of education are aware of the disease. Whereas only 12.9% of illiterate women know about the disease [21]. Regarding transmission routes and measures of prevention, within our study, they were little known and rarely applied. Of the participants who reported recognizing this measurement, the majority were aware that toxoplasmosis can be transmitted through cats. Overall, the literature reports that close contact with cats or cleaning their litter is significantly associated with parasite infection [22,23]. Water and soil contaminated by oocysts represented an important source of contamination; however, only 1.3% of surveyed women knew they should avoid contact with soil and only one woman knew that the toxoplasmosis can be transmitted by water. Pregnant women can also be infected by eating undercooked infected meat, unwashed fruits, or poor hygiene [24].

Lack of hygienic-sanitary measures during meal preparation might lead to the transmission of *T. gondii*. That is why the application of these measures is of great importance for preventing toxoplasmosis. Hence, it is most important that housewives be educated about these preventive practices, as it is they who handling food and preparing meals for their families [25]. However, our study revealed a significant lack of knowledge among housewives related to the disease. In the current study, the participant´s main source of information was their treating physician during pregnancy. According to Jones *et al*. healthcare professionals were the main information source for pregnant women [20]. It is, therefore, recommended implementing an academic intervention for health professionals to enhance their awareness of the disease [26]. The determination of the Toxoplasma-specific antibody status in a pregnant woman during the first trimester of pregnancy is extremely important. Thus, serological surveillance makes it possible to diagnose and manage cases of maternal seroconversion [27]. In Morocco, the Ministry of Health published guidelines in 2006, recommending the systematic screening of toxoplasmosis for a pregnant woman in the first trimester of pregnancy, without any obligation [28]. Congenital toxoplasmosis causes a large public health problem. It represents a significant emerging infectious disease [15], with a worldwide distribution [29]. In our country, there are limited studies concerning the prevalence of CT and seroprevalence of toxoplasmosis [23]. In 2015, 21 infants were confirmed to be born with CT in different Moroccan regional hospital centers, 71% of which were diagnosed at Rabat hospitals [15]. In France, 204 cases of CT were observed in 2012 [30]. The Center for Disease Control and Prevention, estimated annual cases of CT, from 400 to 4000 cases, in the United States [31].

Several studies have demonstrated that screening and treatment of toxoplasmosis during pregnancy results in decreased mother-to-child transmission and clinical sequelae. In the current study, out of all surveyed women, 40% had performed serological testing for toxoplasmosis, among them 41.1% were immunized. Our result is similar to the study performed in Rabat by Laboudi *et al*. which has reported a seroprevalence of 47% among pregnant women [28]. In Algeria, Tunisia, and Libya, the prevalence of latent toxoplasmosis among pregnant women represented, respectively, 44.4%, 44.1%, and 43.4%. In France, the seroprevalence of toxoplasmosis represented 40% in 2003 and 31% in 2016, among women of childbearing age [32]. During primary infection, the woman remains generally asymptomatic. However, she can transmit the infection to the fetus [33]. Therefore, it is the physician's responsibility to explain to the pregnant women, at their first prenatal visit, the importance of serological diagnosis of T. *gondii* infection. Thus, the physician should spend more time with pregnant women to better educate them on how to prevent the disease. Moreover, he should utilize an easy language adapted to the educational level of the interlocutor. Through these measures, many cases of CT may be prevented. Our study's practical implication is the need to improve the knowledge of toxoplasmosis in childbearing and pregnant women regardless of age group, with a particular focus on illiterate women in both rural and urban areas. This might ensure appropriate prevention of further contact with the parasite, especially during pregnancy. Moreover, it is of great importance to generalize the serological test at the onset of the pregnancy. Our survey includes only post-parturient women it may not reflect the whole population. In addition, women in our survey were relatively lower educated and as we found that knowledge about toxoplasmosis increased with educational level; our results may underestimate the knowledge about toxoplasmosis among Moroccan women. Therefore, further studies are needed to assess the toxoplasmosis-related knowledge without specifying whether the woman is parturient or not.

## Conclusion

According to our survey, it is observed that the majority of participants require more information about toxoplasmosis to know the transmission routes, how to prevent infection, and how to avoid the primo-infection in unimmunized women. There is, therefore, necessary to create collaborations between health professionals to establish an education program for women of childbearing age for a better understanding of CT. Furthermore, there is also a need to expand serological screening to detect and monitor seronegative women.

### What is known about this topic

- *In our country, there are limited studies concerning the prevalence of congenital toxoplasmosis and seroprevalence of toxoplasmosis;*
- *Several studies reported a lack of knowledge regarding toxoplasmosis*.

### What this study adds

- *Our study allowed us to understand the awareness and knowledge level of toxoplasmosis among a group of Moroccan women;*
- *There is a need to expand serological screening to detect and monitor seronegative women in our country*.
